# Impact of crystal structure on optical properties and temperature sensing behavior of NaYF_4_:Yb^3+^/Er^3+^ nanoparticles

**DOI:** 10.1039/d3ra03148a

**Published:** 2023-07-11

**Authors:** Charu Dubey, Anjana Yadav, Diksha Baloni, Santosh Kachhap, Sunil Kumar Singh, Akhilesh Kumar Singh

**Affiliations:** a Department of Physical Sciences, Banasthali Vidyapith Banasthali-304022 Rajasthan India akhilesh_singh343@yahoo.com; b Department of Physics, Indian Institute of Technology (Banaras Hindu University) Varanasi-221005 India

## Abstract

We report a comprehensive study of the structural, morphological, and optical properties, and UC-based ratiometric temperature sensing behavior of (α) cubic and (β) hexagonal phases of NaYF_4_:Yb^3+^/Er^3+^ nanoparticles. The α-NaYF_4_:Yb^3+^/Er^3+^ and β-NaYF_4_:Yb^3+^/Er^3+^ nanoparticles were synthesized using co-precipitation and hydrothermal methods, respectively. Powder X-ray diffraction studies confirmed the phase purity of the samples. The morphological studies show uniform particle sizes of both phases; the average particle size of α-NaYF_4_:Yb^3+^/Er^3+^ and β-NaYF_4_:Yb^3+^/Er^3+^ was 9.2 nm and 29 nm, respectively. The Raman spectra reveal five sharp peaks at 253 cm^−1^, 307 cm^−1^, 359 cm^−1^, 485 cm^−1^, and 628 cm^−1^ for β-NaYF_4_:Yb^3+^/Er^3+^, whereas α-NaYF_4_:Yb^3+^/Er^3+^ shows two broad peaks centred at 272 cm^−1^ and 721 cm^−1^. The optical property measurements show that α- and β-NaYF_4_:Yb^3+^/Er^3+^ phases have distinct upconversion emission and temperature sensing behavior. The upconversion emission measurements show that β-NaYF_4_:Yb^3+^/Er^3+^ has higher overall emission intensities and green/red emission intensity ratio. The temperature-dependent upconversion emission measurements show that α-NaYF_4_:Yb^3+^/Er^3+^ has higher energy separation between ^2^H_11/2_ and ^4^S_3/2_ energy states. The temperature sensing performed utilizing these thermally coupled energy levels shows a maximum sensitivity of 0.0069 K^−1^ at 543 K and 0.016 K^−1^ at 422 K for β-NaYF_4_:Yb^3+^/Er^3+^ and α-NaYF_4_:Yb^3+^/Er^3+^, respectively.

## Abstract 2

We report a comprehensive study of the structural, morphological, and optical properties, and UC-based ratiometric temperature sensing behavior of (α) cubic and (β) hexagonal phases of NaYF_4_:Yb^3+^/Er^3+^ nanoparticles.

## Graphical abstract

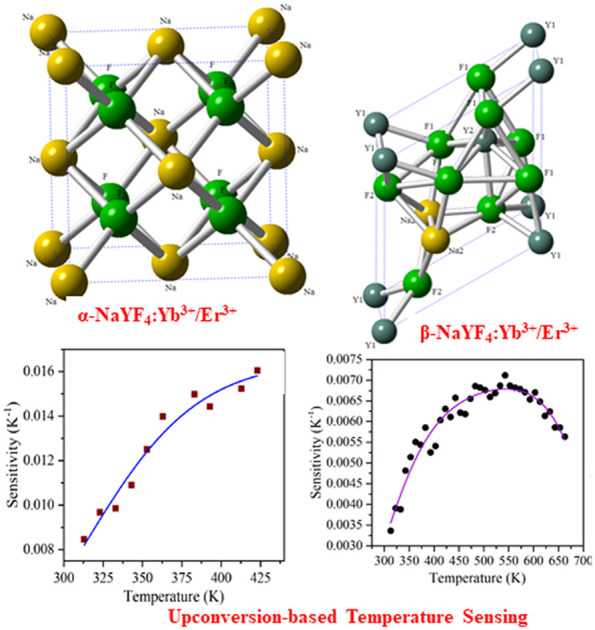

## 1. Introduction

Upconversion (UC) emission is a process where the absorption of two or more low-energy photons by a material leads to the emission of a single photon of higher energy.^1^ This process occurs mainly in lanthanide ions (Er^3+^, Ho^3+^, Tm^3+^, *etc.*) that possess multiple intermediate energy levels with long lifetimes favoring excited state absorption and excited state energy transfer.^2^ There are various mechanisms for the UC process namely excited state absorption (ESA), energy transfer UC (ETU), cooperative energy transfer between pairs of ions, *etc.*^3,4^ The upconverting materials have attracted a lot of interest in bioimaging,^5^ photovoltaics,^6,7^ luminescent security inks,^8,9^ luminescence-based sensing,^10,11^*etc.* The focus of this work would be on temperature sensing using two different phases of an upconverting nanomaterial.

The UC-based temperature sensors utilize the emission intensity ratio originating from two closely spaced energy levels of lanthanide ions that have temperature dependencies.^12^ The advantages of UC-based temperature sensing include high sensitivity, low background noise, and the ability to operate in harsh environments; it is self-referenced (unlike most other thermometers) and can even detect the temperature of nano-dimensional objects.^13,14^ Additionally, upconverting materials can be tailored to operate at specific temperatures, making them suitable for various applications. Overall, UC emission is a promising technology for temperature sensing with potential applications in fields such as materials science, engineering, and medicine.^15^ In literature, Er^3+^, Ho^3+^, and Tm^3+^ doped upconverting materials have been utilized for ratio-metric temperature sensing.^16,17^ Among them, Er^3+^/Yb^3+^ doped upconverting materials have been extensively explored, in recent years, because of their high UC efficiency (in comparison to other activators), suitable energy separation between ^2^H_11/2_ and ^4^S_3/2_ thermally-coupled levels, longer emission lifetime making better signal-to-noise ratio in the temperature measurements, and its biocompatibility making them ideal for use in biological and medical applications.

Here we highlight a brief literature review on Er^3+^/Yb^3+^ doped upconverting materials for temperature sensing. Liu *et al.* studied Er^3+^-doped tellurite glass fiber for temperature sensing and reported that it possesses high sensitivity and linear response over a wide temperature range.^18^ Sachin *et al.* compare the temperature sensing behavior of Yb^3+^/Er^3+^ doped CaMoO_4_ in bulk and nanophosphor and obtained that nanoparticle-based temperature sensors show better results.^19^ Further, they also report that the incorporation of Bi^3+^ in the CaMoO_4_ host reduces the non-radiative channels and creates local symmetry distortion which improves the sensing behavior of Er^3+^ further. Maciel *et al.* studied the role of crystallite size and surrounding medium on temperature sensing behavior of Er^3+^-doped BaTiO_3_; the experiments performed in air, water, and glycerol in physiological temperature range reveals that temperature sensitivity changes with particle size but it does not alter with the surrounding medium.^20^ Mahata *et al.* have shown that the use of Zn^2+^ improved the UC emission intensity and temperature sensing behavior of BaTiO_3_:Er^3+^/Yb^3+^ nanophosphor.^21^ Our group investigated the role of host matrix (Y_2_O_3_, YVO_4_, and YPO_4_) on temperature sensing behavior of Er^3+^ ions and found a very high-temperature sensitivity *i.e.* 0.0105 K^−1^ in YVO_4_ host.^22^

Lanthanide-doped NaYF_4_ exhibits better chemical stability, high UC quantum efficiency, and lower phonon energy than other (*e.g.*, oxide and chloride oxyfluoride) host materials. Cui *et al.* synthesized NaYF_4_:Yb^3+^/Er^3+^ phosphor by hydrothermal process and studied optical temperature sensing behavior, and the maximum sensitivity for β-phase NaYF_4_:Yb^3+^/Er^3+^ were 0.00466 K^−1^ at 550 K.^23^ Tong *et al.* have synthesized spherical NaYF_4_:Yb^3+^/Er^3+^ micro-/nano-crystals *via* microwave-assisted hydrothermal route.^24^ The β-NaYF_4_:Yb^3+^/Er^3+^ show strong green, red, weak blue, and purple UC emissions. The maximum sensitivity for β-NaYF_4_:Yb^3+^/Er^3+^ was 0.0048 K^−1^ at ∼515 K.^24^ Tong *et al.* and Zhang *et al.* have synthesized β-phase NaYF_4_:Sm^3+^/Yb^3+^@NaYF_4_:Er^3+^/Yb^3+^ and NaYF_4_:Er^3+^/Yb^3+^@NaYF_4_:Tm^3+^/Yb^3+^ core–shell nanostructures by thermal decomposition technique, respectively, and studied their temperature sensing behavior.^25,26^ The maximum temperature sensitivity was found to be 0.0046 K^−1^ at 489 K. Their studies showed that the core–shell structure is suitable for accurate temperature detection in the field of photothermal therapy.^26^ As NaYF_4_ has two crystal structures, namely, hexagonal phase (β) and cubic phase (α) that provides different crystallographic environments for Er^3+^ ions and might result in a different UC emission behavior.^27^ Therefore, the objective of this work is to explore the impact of NaYF_4_ (host) crystal structure on the structural, optical, and temperature sensing behavior of Yb^3+^/Er^3+^ doped in α- and β-NaYF_4_. The work is of fundamental importance and would be a proof of concept to explore the sensing behavior of other materials that crystallize in different phases, for the development of high-performance optical temperature sensors.

## 2. Experimental

### 2.1 Materials

Erbium oxide (Er_2_O_3_, 99.99%, Sigma-Aldrich), ytterbium oxide (Yb_2_O_3_, 99.99%, Sigma-Aldrich), yttrium oxide (Y_2_O_3_, 99.9%, Alfa Aesar), ethanol (99.9%, Analytical CS Reagent), sodium hydroxide (NaOH, 99.99% Sigma-Aldrich), 1-octadecene (ODE, 90% technical grade, Sigma-Aldrich), DI water (H_2_O, CDH Pvt. Ltd.), ammonium fluoride (NH_4_F, 95%, Fisher Scientific), oleic acid (OA, 65–88%, Merck), chloroform (CHCl_3_, 99% Sisco Research Laboratories Pvt. Ltd), and hydrochloric acid (HCl, 37% Sigma-Aldrich), methanol (99%, Ranken Chemicals) were used at raw materials for synthesizing NaYF_4_:Yb^3+^/Er^3+^ phosphor without any further purification.

### 2.2 Synthesis of α-NaYF_4_:Yb^3+^/Er^3+^ by co-precipitation method

The α-NaYF_4_:Yb^3+^/Er^3+^ was synthesized by the co-precipitation method.^28^ The stoichiometric amount of Y_2_O_3_, Yb_2_O_3_, and Er_2_O_3_ was dissolved in dilute HCl for synthesizing α-NaYF_4_:Yb^3+^/Er^3+^ (α-NaY_0.78_Yb_0.2_Er_0.02_F_4_). In a 100 mL flask with 16 mL oleic acid and 24 mL 1-octadecene, 1.6 mmol of rare earth chloride was added as an aqueous solution. To remove the solution's moisture content, the reaction mixture was heated at 423 K for 30 min while being continuously dry N_2_ purged, and then it was allowed to cool at ambient temperature (300 K). The aforementioned solution was then supplemented with 4.8 mmol NH_4_F and 1.6 mmol NaOH dissolved in 20 mL of methanol, and stirred for 30 minutes. The reaction mixture was heated at 350 K under constant dry N_2_ purging until the entire methanol had evaporated (around 1.5 hours). The reaction mixture was heated to 513 K for 45 min while being continuously purged with dry N_2_. After that, it was cooled to room temperature. By adding ethanol to the reaction mixture, the synthesized nanoparticles were precipitated and then collected by centrifugation. The nanoparticles were repeatedly cleaned in ethanol before being dispersed in chloroform. In the co-precipitation method the reaction temperature, reaction time, and ligand molecules control the phase purity and size/morphology of the synthesized upconverting nanoparticles. Kavand *et al.* have reported that for the co-precipitation process carried out in a round bottom flask at 573 K β nucleation starts after 30 min.^29^ Chen and Wang have shown that the introduction of oleylamine regulates phase transformation from the α-phase to the β-phase and also reduces the required temperature for phase transformation.^30^

### 2.3 Synthesis of β-NaYF_4_:Yb^3+^/Er^3+^ by hydrothermal method

To synthesize β-NaYF_4_:Yb^3+^/Er^3+^ (β-NaY_0.78_Yb_0.2_Er_0.02_F_4_) by hydrothermal method, 7.5 mL DI water solution of 1.5 g NaOH was mixed with 25 mL of ethanol and 25 mL of oleic acid under stirring. To the resulting mixture, 10 mL aqueous solution of lanthanide chloride (0.5735 g YCl_3_, 0.1616 g YbCl_3_, and 0.01333 g ErCl_3_) and 5 mL methanol solution of 0.3741 g NH_4_F were added while stirring. In 100 mL of Teflon-lined autoclave, the solution was transferred, and it was heated at 473 K for 5 hours. The resulting phosphor was centrifuged, collected, and then cleaned multiple times with ethanol and DI water before being redispersed in cyclohexane. The advantage of the hydrothermal process is that it requires lower temperature, a one-step synthetic procedure, environmental friendliness, good phase control, and uniform particle size.^31^

### 2.4 Characterization

For structural analysis, powder samples were characterized using Bruker D8 Advance X-ray diffractometer operating at 40 kV tube voltage and 40 mA current in the 2*θ* range 10 to 90°. The Tecnai G2 TWIN was used to take TEM pictures of the NaYF_4_:Yb^3+^/Er^3+^ phosphor while operating at 200 kV acceleration voltages. The Raman spectra of NaYF_4_:Yb^3+^/Er^3+^ phosphor were recorded using a Thermo Scientific DXRxi Raman imaging microscope instrument equipped with a 532 nm laser. To capture the photoluminescence (PL) excitation and emission spectra of the powder sample, a fluoromax-plus spectrofluorometer with a 150 W xenon flash lamp was employed (without using an integrating sphere). PL decay measurements were carried out at the same set-up using a pulsed xenon lamp (25 W). The 976 nm tunable continuous-wave diode laser was used as an external excitation source in the same setup to record the UC emissions. A homemade heater equipped with a k-type thermocouple was utilized for the temperature-dependent UC emission measurements, and it was placed right next to the laser's focal point. A variac was used to control the temperature of the (by controlling the voltage of the heater) sample.

## 3. Results and discussions

### 3.1 Crystal structure

**Fig. 1:**
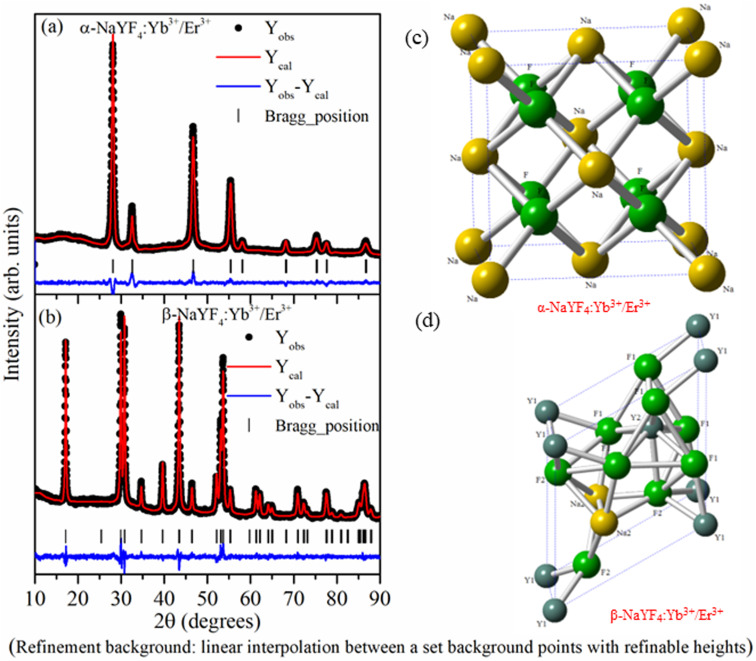
Rietveld refinement of (a) α-NaYF_4_:Yb^3+^/Er^3+^ and (b) β-NaYF_4_:Yb^3+^/Er^3+^ using *F*_*m*3̄*m*_ and *P*_6̄_ space group, respectively. The experimental, calculated, and difference profiles are represented by black dots, red lines, and blue (bottom) lines, respectively. Bragg reflections are denoted by vertical tick marks above the difference profile. (c) and (d) shows the crystal structure of α-NaYF_4_:Yb^3+^/Er^3+^ and β-NaYF_4_:Yb^3+^/Er^3+^ drawn based on the Rietveld refinement data.

As the crystallographic structure and phase purity play an important role in the UC emission intensity, the powder X-ray diffraction data of NaYF_4_:Yb^3+^/Er^3+^ nanophosphor prepared using co-precipitation and hydrothermal methods were recorded. The Rietveld refinement using the FullProf software was used to analyze powder X-ray data. The calculated intensity profile was compared with experimental data and the best fit was obtained by the least squares fitting method. The *R*-factor (residual functions), which is an indicator of the quality of fit, is minimized to attain the best refinement. Fig. 1 reveals that all the XRD peaks in the sample synthesized by co-precipitation and hydrothermal methods were indexed considering the *F*_*m*3̄*m*_ space group of cubic (α-NaYF_4_:Yb^3+^/Er^3+^ phase) and *P*_6̄_ space group of hexagonal (β-NaYF_4_:Yb^3+^/Er^3+^) crystal systems, respectively. There is no impurity phase or overlapping of α- and β-NaYF_4_:Yb^3+^/Er^3+^ phases.

In the α-NaYF_4_:Yb^3+^/Er^3+^ phase, 4a crystallographic sites of *F*_*m*3̄*m*_ space group is occupied by Na^+^ and Y^3+^/Yb^3+^/Er^3+^ cations in a 1 : 1 ratio, whereas F^−^ occupies the 8c crystallographic sites. In the β-NaYF_4_:Yb^3+^/Er^3+^ phase, F^−^ occupies 3j and 3k crystallographic sites. Metal cations occupy three different crystallographic sites. Out of three sites, 1a and 1f are 9-fold coordinated with fluoride ions forming a tricapped trigonal prism. The 1a site is occupied by Y^3+^/Yb^3+^/Er^3+^ cations, and the 1f site is occupied by Na^+^ and Y^3+^/Yb^3+^/Er^3+^ cations in a 1 : 1 ratio. Within the distorted octahedral, formed by six fluoride anions, the half of 2h site is occupied by Na^+^ and half of the sites are vacant. Fig. 1(c) and (d) show the crystal structure of α-NaYF_4_:Yb^3+^/Er^3+^ and β-NaYF_4_:Yb^3+^/Er^3+^ phosphor based on the Rietveld refinement parameters. Table 1 shows the atomic positions of different atoms, the occupancy factor of different crystallographic sites, lattice parameters, and other refined parameters.

**Table 1:** Refine structural parameters for α-NaYF_4_:Yb^3+^/Er^3+^ (space group: *F*_*m*3̄*m*_), β-NaYF_4_:Yb^3+^/Er^3+^ (space group: *P*_6̄_) nanophosphors

Phase	Atoms	Positional coordinates			Occupancy	Multiplicity
		*X*	*Y*	*Z*		
α-Phase	Na	0.0000(0)	0.0000(0)	0.0000(0)	0.01	4a
	Y/Yb/Er	0.0000(0)	0.0000(0)	0.0000(0)	0.01	4a
	F	0.2500(0)	0.2500(0)	0.2500(0)	0.042	8c
*a* = *b* = *c* = 5.5010 (1) Å, *R*_p_ = 3.59, *R*_wp_ = 4.80, *R*_exp_ = 12.6, *χ*^2^ = 3.42						
β-Phase	Na1	0.6666(0)	0.3333(0)	0.5000(0)	0.083	1f
	Na2	0.3333(0)	0.6666(0)	0.8641(24)	0.167	2h
	Y1	0.0000(0)	0.0000(0)	0.0000(0)	0.167	1a
	Y2	0.6666(0)	0.3333(0)	0.5000(0)	0.083	1f
	F1	0.6669(0)	0.07814(18)	0.0000(0)	0.333	3j
	F2	0.7107(17)	0.7606(18)	0.5000(0)	0.333	3k
*a* = *b* = 5.9752(2) Å, *c* = 3.5086(2) Å, *R*_p_ = 4.34, *R*_wp_ = 5.78, *R*_exp_ = 2.87, *χ*^2^ = 4.06						

### 3.2 Morphology

**Fig. 2:**
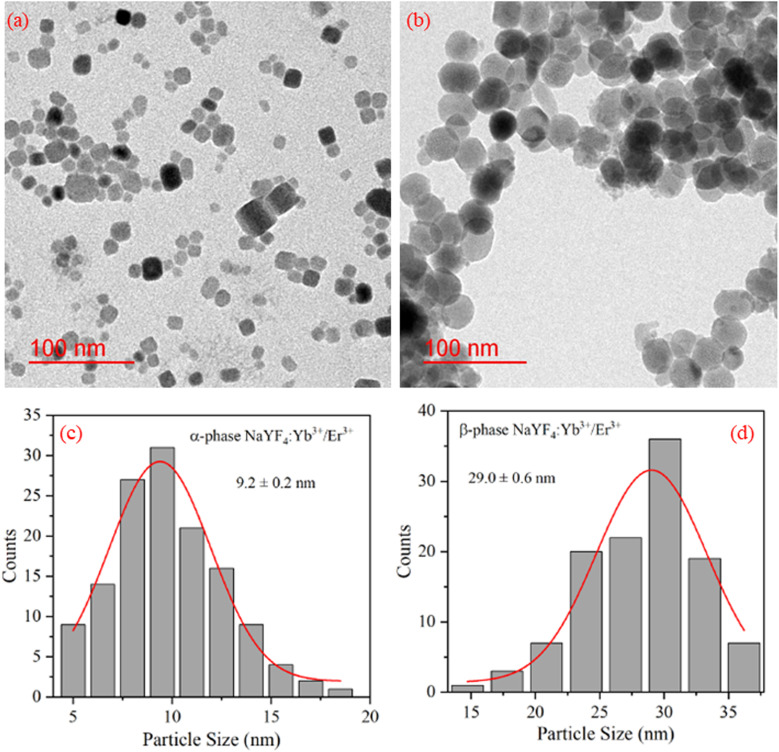
TEM image of (a) α-NaYF_4_:Yb^3+^/Er^3+^, (b) β-NaYF_4_:Yb^3+^/Er^3+^ nanophosphor. (c) and (d) shows the particle size distribution of α-NaYF_4_:Yb^3+^/Er^3+^, and β-NaYF_4_:Yb^3+^/Er^3+^ nanophosphor, respectively.

The TEM images of NaYF_4_:Yb^3+^/Er^3+^ nanophosphor synthesized using co-precipitation (α-phase) and hydrothermal (β-phase) methods were captured for morphological studies and to analyze particle size. Fig. 2(a and b) reveals the cubic and spherical morphology of α-NaYF_4_:Yb^3+^/Er^3+^ and β-NaYF_4_:Yb^3+^/Er^3+^, respectively. The sample synthesized using the hydrothermal method shows a bigger particle size than synthesized using the co-precipitation method. The particle sizes were quite uniform. To determine the average particle size from TEM images ∼150 particles were evaluated. A Gaussian fit to the histogram shown in Fig. 2(c and d) indicates the average particle size of α-NaYF_4_:Yb^3+^/Er^3+^ and β-NaYF_4_:Yb^3+^/Er^3+^ as 9.2 nm and 29 nm, respectively.

### 3.3 Raman studies

**Fig. 3:**
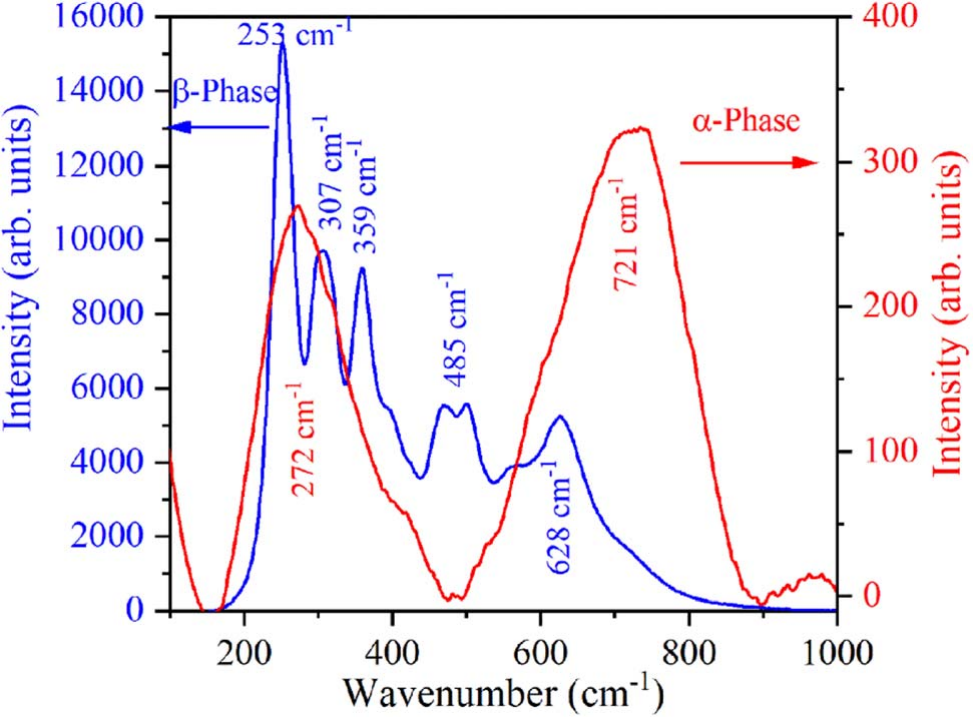
Raman spectra of α-NaYF_4_:Yb^3+^/Er^3+^ and β-NaYF_4_:Yb^3+^/Er^3+^ nanophosphors.

The Raman spectrum of β-NaYF_4_:Yb^3+^/Er^3+^ shown in Fig. 3 reveals five Raman peaks at 253 cm^−1^, 307 cm^−1^, 359 cm^−1^, 485 cm^−1^, and 628 cm^−1^. Similar Raman spectra were also reported in the literature for β-NaYF_4_:Yb^3+^/Er^3+^.^32,33^ Klier and Kumke observed Raman peaks at 251 cm^−1^, 303 cm^−1^, 359 cm^−1^, 492 cm^−1^, and 625 cm^−1^.^32^ Shan *et al.* have attributed Raman peaks observed at 485 cm^−1^, and 628 cm^−1^ to organic surfactant present at the nanoparticle surfaces.^33^ Further in β-NaYF_4_:Yb^3+^/Er^3+^ the highest phonon energy is 500 cm^−1^ which makes it an excellent host for UC.^34,35^ The Raman spectrum of α-NaYF_4_:Yb^3+^/Er^3+^ shows two broad peaks centered at 272 cm^−1^ and 721 cm^−1^. Broad Raman band above 500 cm^−1^ is the signature of α-NaYF_4_:Yb^3+^/Er^3+^.^34,36^

### 3.4 Down-shifting emission

**Fig. 4:**
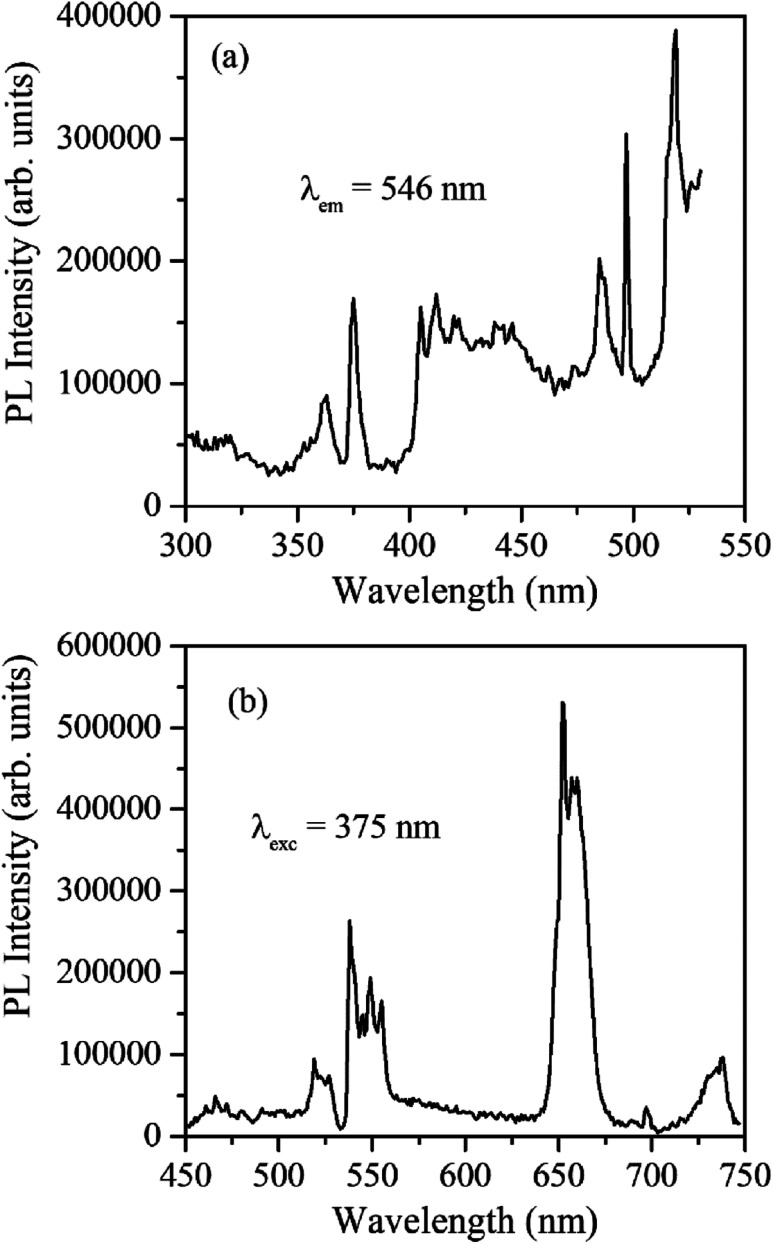
(a) PL excitation spectrum of β-NaYF_4_:Yb^3+^/Er^3+^ monitored at 546 nm emission wavelength. (b) The PL emission spectrum of β-NaYF_4_:Yb^3+^/Er^3+^ was monitored at 375 nm excitation wavelength.

**Fig. 5:**
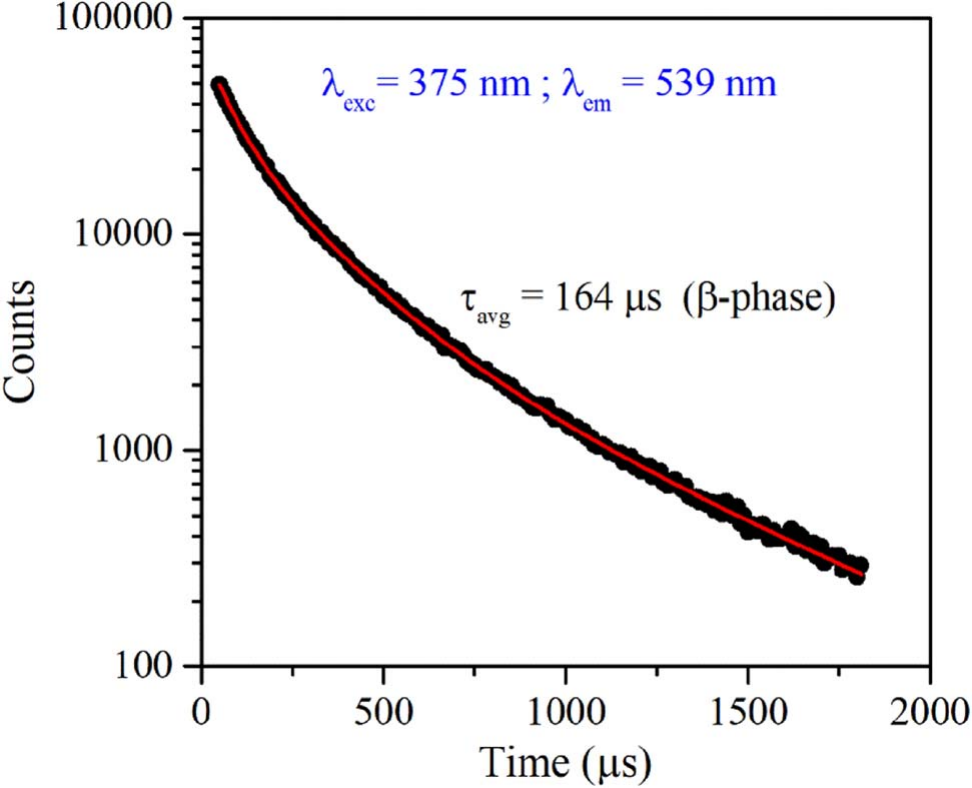
Time-resolved PL of β-NaYF_4_:Yb^3+^/Er^3+^ phosphor monitored at 375 nm excitation and 539 nm emission wavelength, respectively.

Fig. 4 shows the excitation and emission spectrum of β-NaYF_4_:Yb^3+^/Er^3+^ monitored at 546 nm emission and 375 nm excitation wavelengths, respectively. The excitation spectrum shows peaks at 362 nm, 375 nm, 404 nm, 487 nm, and 518 nm that correspond to ^4^G_9/2_ ← ^4^I_15/2_, ^4^G_11/2_ ← ^4^I_15/2_, ^2^H_9/2_ ← ^4^I_15/2_, ^4^F_7/2_ ← ^4^I_15/2_, and ^2^H_11/2_ ← ^4^I_15/2_ transitions of Er^3+^ ions, respectively. The down-shifting emission intensities in β-NaYF_4_:Yb^3+^/Er^3+^ are quite weak that further diminish in the α-NaYF_4_:Yb^3+^/Er^3+^ nanophosphor and it was not recordable. The PL decay of the green emission band in β-NaYF_4_:Yb^3+^/Er^3+^ was monitored at 375 nm excitation and 539 nm emission wavelengths, respectively, shown in Fig. 5. The average lifetime determined by the tri-exponential fit was found to be 164 μs.

### 3.5 UC emission

**Fig. 6:**
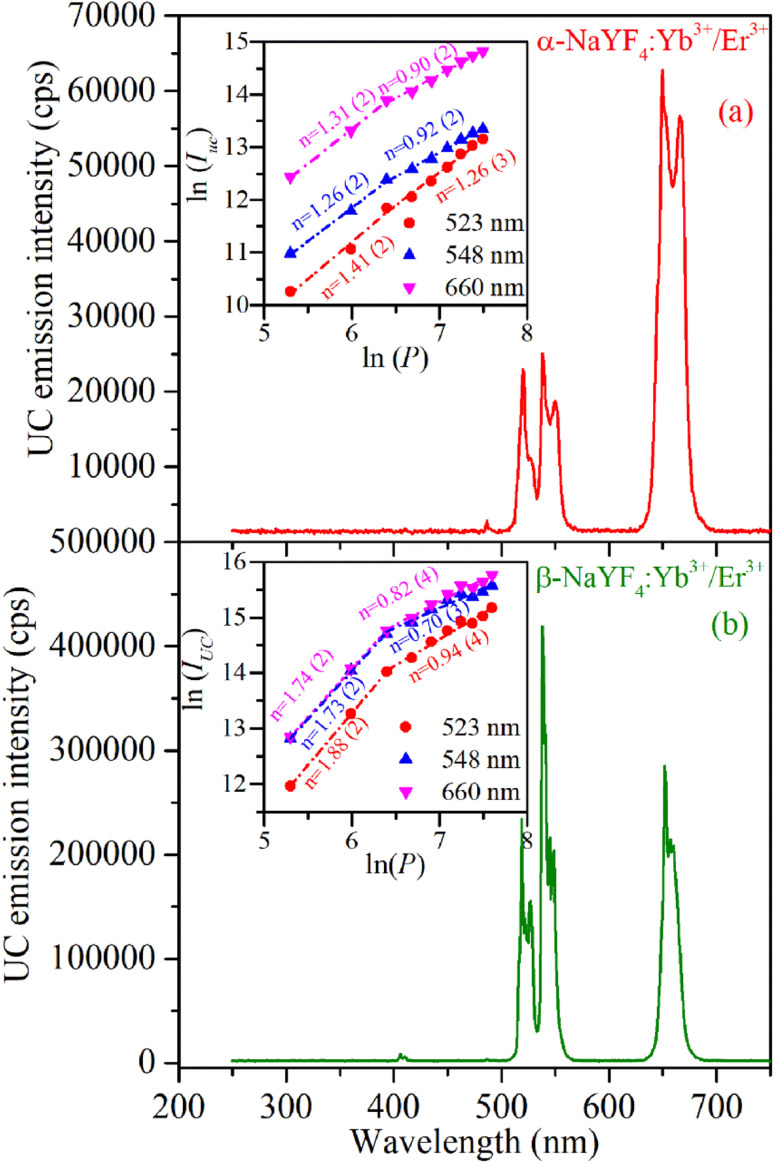
UC emission spectra of (a) α-NaYF_4_:Yb^3+^/Er^3+^ and (b) β-NaYF_4_:Yb^3+^/Er^3+^ were recorded using continuous-wave diode laser radiating at 976 nm wavelength. Inset to the Figures shows ln(*I*) and ln(*P*) plot at different emission wavelengths.

The UC emission spectra of α-NaYF_4_:Yb^3+^/Er^3+^ and β-NaYF_4_:Yb^3+^/Er^3+^ (shown in Fig. 6) were recorded using a 976 nm wavelength CW diode laser. The 976 nm wavelength photons absorbed by the Er^3+^ ions in its ground state result in the resonant excitation of the ^4^I_11/2_ level, moreover it is resonant with ^2^F_7/2_ → ^2^F_5/2_ transition of Yb^3+^ which has high absorption cross section than Er^3+^ ions and works as a sensitizer to Er^3+^ ions.^37^ The α-NaYF_4_:Yb^3+^/Er^3+^ shows emission bands at 523, 548, and 660 nm corresponding to the ^2^H_11/2_ → ^4^I_15/2_, ^4^S_3/2_ → ^4^I_15/2_ and ^4^F_9/2_ → ^4^I_15/2_ transitions of Er^3+^ ion, respectively. The β-NaYF_4_:Yb^3+^/Er^3+^ along with these emission bands show an additional emission band at 407 nm corresponding to the ^2^H_9/2_ → ^4^I_15/2_ transition of Er^3+^ ions. The comparison of UC emission reveals that the overall emission intensity of β-NaYF_4_:Yb^3+^/Er^3+^ is 4.6 times higher than that of α-NaYF_4_:Yb^3+^/Er^3+^. Furthermore, in β-NaYF_4_:Yb^3+^/Er^3+^ the intensity ratio of green to red emission bands is 1.437 whereas it is 0.376 in α-NaYF_4_:Yb^3+^/Er^3+^ resulting in greenish and reddish emission color in β-NaYF_4_:Yb^3+^/Er^3+^ and α-NaYF_4_:Yb^3+^/Er^3+^, respectively. The difference in the UC emission behavior might be because of higher phonon frequency and different crystallographic environments for Er^3+^ ions in α-NaYF_4_:Yb^3+^/Er^3+^. The distinct Stark splitting in the emission bands is induced by the crystal field of ligands around the Er^3+^ ions in the host lattice.

As the UC is a non-linear optical process the emission intensities of various bands are proportional to the ‘*n*^th^’ power of laser excitation power.

1 *I*_UC_ ∝ *P*^*n*^

where *n* is the number of absorbed photons per emitted photon in the UC process and it can be obtained from the slope of ln(*I*) *versus* ln(*P*) plot. The inset to the Fig. 6 shows that in β-NaYF_4_:Yb^3+^/Er^3+^ the ^2^H_11/2_ → ^4^I_15/2_, ^4^S_3/2_ → ^4^I_15/2_ and ^4^F_9/2_ → ^4^I_15/2_ transitions involve two photons as the value of ‘*n*’ is 1.88, 1.73 and 1.74, respectively. The value of ‘*n*’ decreases at higher laser power which could be because of cross-relaxation processes resulting in saturation of UC intensity. The schematic energy level diagram involving various energy states and mechanisms for the UC emission in the Er^3+^ ions is reported in our previous work.^22^ The value of ‘*n*’ for these transitions in α-NaYF_4_:Yb^3+^/Er^3+^ (for ^2^H_11/2_ → ^4^I_15/2_, ^4^S_3/2_ → ^4^I_15/2_ and ^4^F_9/2_ → ^4^I_15/2_ transitions 1.41, 1.26, and 1.31, respectively) is significantly lower than in β-NaYF_4_:Yb^3+^/Er^3+^ phosphor. This might be because of different phonon coupling in the cubic and hexagonal lattices.

### 3.6 Temperature sensing

The ^2^H_11*/*2_ and ^4^S_3*/*2_ energy levels in the Er^3+^ ions are thermally coupled to each other, an increase in the temperature results in more population to the ^2^H_11*/*2_ energy level. This in turn reflected in a change in the emission intensity ratio of ^2^H_11/2_ → ^4^I_15/2_ and ^4^S_3/2_ → ^4^I_15/2_ transitions of Er^3+^ ions centered at 523 nm and 548 nm, respectively. In the temperature-dependent UC emission measurements, the relative populations of ^2^H_11/2_ and ^4^S_3/2_ energy levels can be expressed by the Boltzmann distribution function that leads to,2
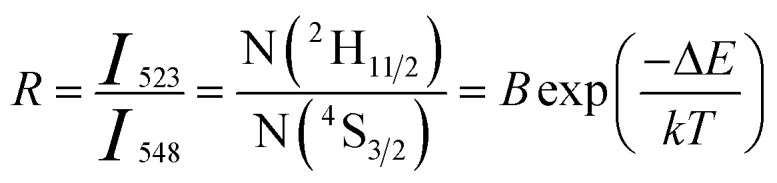
where, *I*_523_ and *I*_548_ are the integrated intensity of the ^2^H_11/2_ → ^4^I_15/2_ (515–540 nm) and ^4^S_3/2_ → ^4^I_15/2_ (540–565 nm) emission bands, N(^2^H_11/2_) and N(^4^S_3/2_) are population in ^2^H_11/2_, ^4^S_3/2_ energy states, *B* is a pre-exponential constant, *k* is the Boltzmann constant, *T* is the absolute temperature, and Δ*E* is the energy difference between the ^2^H_11/2_ and ^4^S_3/2_ thermally coupled levels of Er^3+^ ions.

**Fig. 7:**
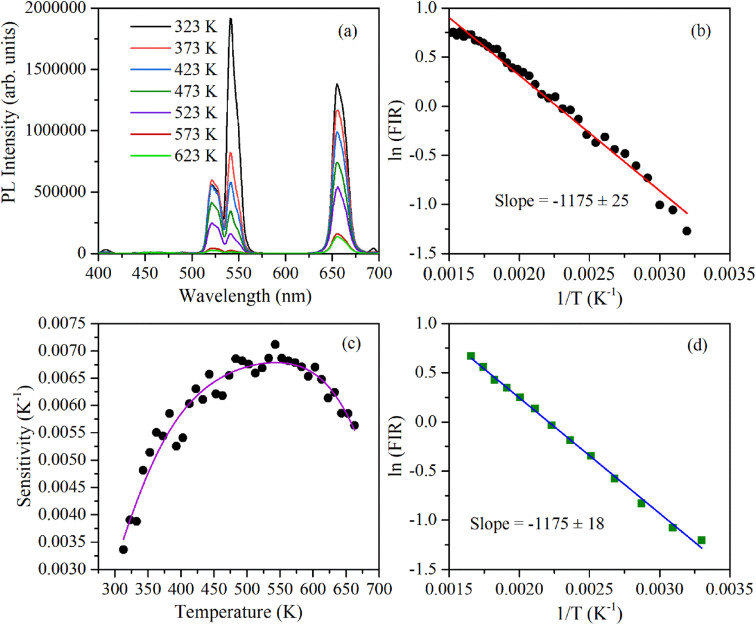
UC-based ratiometric temperature sensing using β-NaYF_4_:Yb^3+^/Er^3+^ phosphor: (a) UC emission spectra measured between 300 and 650 K, (b) plot of ln(FIR) (using 523 and 548 nm emission) as a function of the inverse absolute temperature during heating, (c) temperature sensitivity as a function of temperature, (d) plot of ln(FIR) as a function of the inverse absolute temperature during cooling.

**Fig. 8:**
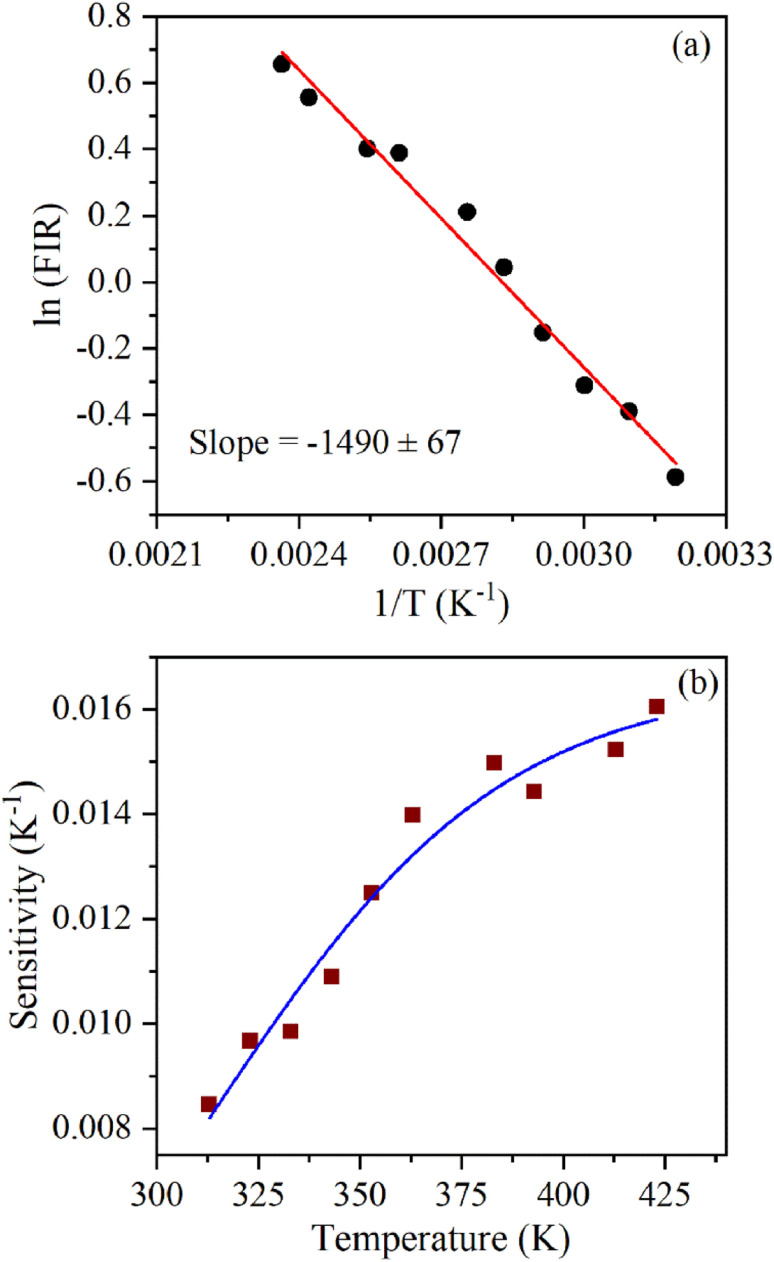
UC-based ratiometric temperature sensing using α-NaYF_4_:Yb^3+^/Er^3+^ nanophosphor: (a) plot of ln(FIR) (using 523 and 548 nm emission) as a function of the inverse absolute temperature, (b) temperature sensitivity as a function of temperature.

To calculate the temperature sensitivity we take the natural logarithm of expression (2),3
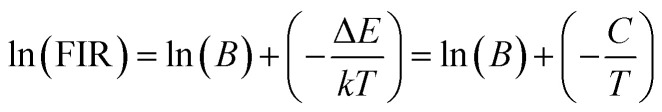
where, *C* is slope of the ln(FIR) *versus* 1/*T* plot. The temperature sensitivity can be defined as the rate of change of FIR with temperature and is given as,4
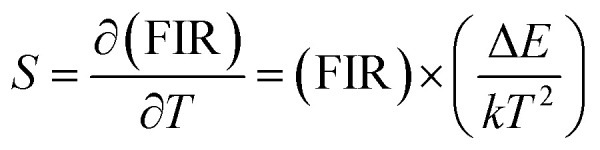
In the literature, the UC emission intensity ratio of 523 nm and 548 nm emission bands as a function of temperatures was explored for realizing ratiometric temperature sensing. The objective of this work (as indicated above) was to study/compare the temperature-sensing behavior of α- and β-phases of NaYF_4_:Yb^3+^/Er^3+^ phosphor. Fig. 7(a) shows the UC emission spectra of β-NaYF_4_:Yb^3+^/Er^3+^ at different temperatures excited using a 976 nm continuous-wave diode laser radiating at 1.2 W. By comparing the emission intensities of both the green emission bands it can be observed that population in 523 nm emission bands increases and 548 nm emission band decreases with increase in the temperature of the sample. Also, by observing the emission intensities at 660 nm it can be inferred that the overall UC intensities decrease significantly with an increase in the temperature of the sample. The change in the population of 523 nm and 548 nm emission bands can be more clearly observed by the ln(FIR) *versus* 1/*T* plot (during heating) shown in Fig. 7(b). Fig. 7(c) shows temperature sensitivity as a function of temperature computed using expression (4). The maximum sensitivity is found to be 0.069 K^−1^ at 543 K. Above this temperature, temperature sensitivity decreases. This might be because of the population saturation of the ^2^H_11/2_ level at higher temperatures. This value of temperature sensitivity is comparable to the reported temperature sensitivity for UC emission-based temperature sensing in different hosts.^38–40^Fig. 7(d) shows ln(FIR) *versus* 1/*T* plot during a cooling run which has the same slope as observed during the heating. This indicates good reproducibility of temperature sensing measurements and good temperature stability of nanophosphor. Fig. 8(a) shows ln(FIR) *versus* 1/*T* plot for α-NaYF_4_:Yb^3+^/Er^3+^ during a heating run. The value of the slope is larger in this indicating higher temperature sensitivity for α-NaYF_4_:Yb^3+^/Er^3+^ nanophosphor. The temperature sensitivity as a function of temperature plotted for α-NaYF_4_:Yb^3+^/Er^3+^ phosphor shows maximum sensitivity of 0.016 K^−1^ at 422 K.

The energy separation between ^2^H_11/2_ and ^4^S_3/2_ thermally coupled levels of Er^3+^ ions were calculated for α- and β-NaYF_4_:Yb^3+^/Er^3+^ using slope obtained in Fig. 7(b) and 8(a) and it was observed 816 cm^−1^ and 1035 cm^−1^, respectively. This could also be a reason for the distinct temperature sensitivity of α- and β-phases of NaYF_4_:Yb^3+^/Er^3+^. As overall UC emission intensity for α-NaYF_4_:Yb^3+^/Er^3+^ was less in comparison to β-NaYF_4_:Yb^3+^/Er^3+^ it could not be explored for a wide temperature range. However, because of biocompatibility, small particle size and high sensitivity α-NaYF_4_:Yb^3+^/Er^3+^ phosphor could be a good choice for temperature sensing at the physiological range in biological applications.^5,41,42^

## 4. Conclusion

Phase pure α- (cubic) and β-NaYF_4_:Yb^3+^/Er^3+^ (hexagonal) nanoparticles were synthesized. Both the phases show uniform morphology with an average particle size of 9.2 nm and 29 nm for α-NaYF_4_:Yb^3+^/Er^3+^ and β-NaYF_4_:Yb^3+^/Er^3+^, respectively. The Raman studies show completely different behavior for both phases. The UC studies show that the green-to-red emission intensity ratio is 1.437 and 0.376 in β-NaYF_4_:Yb^3+^/Er^3+^ and α-NaYF_4_:Yb^3+^/Er^3+^, respectively. Temperature sensing performed utilizing the fluorescence intensity ratio of ^2^H_11/2_ and ^4^S_3/2_ levels of Er^3+^ ions shows higher maximum sensitivity (0.016 K^−1^) for α-NaYF_4_:Yb^3+^/Er^3+^ than (0.069 K^−1^) β-NaYF_4_:Yb^3+^/Er^3+^ nanoparticles. The obtained results suggest that β-NaYF_4_:Yb^3+^/Er^3+^ could be promising for temperature sensing in a wide temperature range, whereas α-NaYF_4_:Yb^3+^/Er^3+^ could be used for physiological range in biological applications.

## Author contributions

Charu Dubey: acquisition of data, analysis and/or interpretation of data, drafting the manuscript, revising the manuscript. Anjana Yadav: acquisition of data, analysis and/or interpretation of data. Diksha Baloni: acquisition of data, drafting the manuscript. Santosh Kachhap: Rietveld refinement and structural analysis, Sunil Kumar Singh: analysis and/or interpretation of data, revising the manuscript. Akhilesh Kumar Singh: conceptualization, analysis and/or interpretation of data, drafting the manuscript, revising the manuscript.

## Conflicts of interest

There are no conflicts to declare.

## Supplementary Material

## References

[cit1] Zhou J., Liu Q., Feng W., Sun Y., Li F. (2015). Chem. Rev..

[cit2] Auzel F. (2004). Chem. Rev..

[cit3] Dong H., Sun L.-D., Yan C.-H. (2013). Nanoscale.

[cit4] Catunda T., Nunes L. A. O., Florez A., Messaddeq Y., Aegerter M. A. (1996). Phys. Rev. B.

[cit5] Rai M., Singh S. K., Singh A. K., Prasad R., Koch B., Mishra K., Rai S. B. (2015). ACS Appl. Mater. Interfaces.

[cit6] Shalav A., Richards B. S., Green M. A. (2007). Sol. Energy Mater. Sol. Cells.

[cit7] Singh A. K., Singh S. K., Kumar P., Gupta B. K., Prakash R., Rai S. B. (2014). Sci. Adv. Mater..

[cit8] Dubey C., Yadav A., Baloni D., Singh S., Singh A. K., Singh S. K., Singh A. K. (2023). Methods Appl. Fluoresc..

[cit9] Shahi P. K., Singh P., Singh A. K., Singh S. K., Rai S. B., Prakash R. (2017). J. Colloid Interface Sci..

[cit10] Monika R. S. Y., Bahadur A., Rai S. B. (2019). RSC Adv..

[cit11] Pandey A., Rai V. K., Kumar V., Kumar V., Swart H. C. (2015). Sens. Actuators, B.

[cit12] Singh A. K., Singh S. K., Gupta B. K., Prakash R., Rai S. B. (2013). Dalton Trans..

[cit13] Vetrone F., Naccache R., Zamarrón A., Juarranz de la Fuente A., Sanz-Rodríguez F., Martinez Maestro L., Martín Rodriguez E., Jaque D., García Solé J., Capobianco J. A. (2010). ACS Nano.

[cit14] Lin G., Jin D. (2021). ACS Sens..

[cit15] Chen J., Zhao J. X. (2012). Sensors.

[cit16] Yadav R. S., Dhoble S. J., Rai S. B. (2018). Sens. Actuators, B.

[cit17] Pandey A., Rai V. K. (2013). Dalton Trans..

[cit18] Liu L., Sun Z., Ma C., Tao R., Zhang J., Li H., Zhao E. (2018). Mater. Res. Bull..

[cit19] Singh S., Kachhap S., Singh A. K., Pattnaik S., Singh S. K. (2022). Methods Appl. Fluoresc..

[cit20] Maciel G. S., Alencar M. A. R. C., de Araújo C. B., Patra A. (2010). J. Nanosci. Nanotechnol..

[cit21] Mahata M. K., Koppe T., Mondal T., Brüsewitz C., Kumar K., Kumar Rai V., Hofsäss H., Vetter U. (2015). Phys. Chem. Chem. Phys..

[cit22] Singh A. K., Shahi P. K., Rai S. B., Ullrich B. (2015). RSC Adv..

[cit23] Cui Y., Meng Q., Lü S., Sun W. (2019). ChemistrySelect.

[cit24] Tong L., Li X., Hua R., Li X., Zheng H., Sun J., Zhang J., Cheng L., Chen B. (2015). J. Lumin..

[cit25] Tong L., Li X., Zhang J., Xu S., Sun J., Zheng H., Zhang Y., Zhang X., Hua R., Xia H., Chen B. (2017). Opt. Express.

[cit26] Zhang Y., Xu S., Li X., Zhang J., Sun J., Xia H., Hua R., Chen B. (2018). Opt. Mater. Express.

[cit27] Leménager G. A.-O., Tusseau-Nenez S., Thiriet M., Coulon P. E., Lahlil K., Larquet E., Gacoin T. A.-O. (2019). Nanomaterials.

[cit28] Geitenbeek R. G., Prins P. T., Albrecht W., van Blaaderen A., Weckhuysen B. M., Meijerink A. (2017). J. Phys. Chem. C.

[cit29] Kavand A., Serra C. A., Blanck C., Lenertz M., Anton N., Vandamme T. F., Mély Y., Przybilla F., Chan-Seng D. (2021). ACS Appl. Nano Mater..

[cit30] Chen B., Wang F. (2020). Inorg. Chem. Front..

[cit31] Yan C., Zhao H., Perepichka D. F., Rosei F. (2016). Small.

[cit32] Klier D. T., Kumke M. U. (2015). J. Mater. Chem. C.

[cit33] Shan J., Uddi M., Yao N., Ju Y. (2010). Adv. Funct. Mater..

[cit34] Modak M. D., Damarla G., Maity S., Chaudhary A. K., Paik P. (2019). RSC Adv..

[cit35] Renero-Lecuna C., Martín-Rodríguez R., Valiente R., González J., Rodríguez F., Krämer K. W., Güdel H. U. (2011). Chem. Mater..

[cit36] Assaaoudi H., Shan G.-B., Dyck N., Demopoulos G. P. (2013). CrystEngComm.

[cit37] Talewar R. A., Mahamuda S., Swapna K., Venkateswarlu M., Rao A. S. (2021). Mater. Res. Bull..

[cit38] Pattnaik S., Rai V. K. (2020). Mater. Res. Bull..

[cit39] Siaï A., Haro-González P., Horchani Naifer K., Férid M. (2018). Opt. Mater..

[cit40] Voiculescu A. M., Hau S., Stanciu G., Avram D., Gheorghe C. (2022). J. Lumin..

[cit41] Liu Y., Tu D., Zhu H., Chen X. (2013). Chem. Soc. Rev..

[cit42] Yi Z., Li X., Xue Z., Liang X., Lu W., Peng H., Liu H., Zeng S., Hao J. (2015). Adv. Funct. Mater..

